# Clinical Utility of Multimodality Imaging with Dynamic Contrast-Enhanced MRI, Diffusion-Weighted MRI, and ^18^F-FDG PET/CT for the Prediction of Neck Control in Oropharyngeal or Hypopharyngeal Squamous Cell Carcinoma Treated with Chemoradiation

**DOI:** 10.1371/journal.pone.0115933

**Published:** 2014-12-22

**Authors:** Shu-Hang Ng, Chien-Yu Lin, Sheng-Chieh Chan, Yu-Chun Lin, Tzu-Chen Yen, Chun-Ta Liao, Joseph Tung-Chieh Chang, Sheung-Fat Ko, Hung- Ming Wang, Chee-Jen Chang, Jiun-Jie Wang

**Affiliations:** 1 Molecular Imaging Center, Chang Gung Memorial Hospital, Chang Gung University, Kueishan, Taoyuan, Taiwan; 2 Department of Diagnostic Radiology, Chang Gung Memorial Hospital, Chang Gung University, Kueishan, Taoyuan, Taiwan; 3 Department of Medical Imaging and Radiological Sciences, Chang Gung Memorial Hospital, Chang Gung University, Kueishan, Taoyuan, Taiwan; 4 Department of Radiation Oncology, Chang Gung Memorial Hospital, Chang Gung University, Kueishan, Taoyuan, Taiwan; 5 Department of Nuclear Medicine, Chang Gung Memorial Hospital, Chang Gung University, Kueishan, Taoyuan, Taiwan; 6 Department of Otorhinolaryngology, Head and Neck Surgery, Chang Gung Memorial Hospital, Chang Gung University, Kueishan, Taoyuan, Taiwan; 7 Department of medical Oncology, Chang Gung Memorial Hospital, Chang Gung University, Kueishan, Taoyuan, Taiwan; 8 Clinical Informatics and Medical Statistics Research Center, Chang Gung Memorial Hospital, Chang Gung University, Kueishan, Taoyuan, Taiwan; The University of Hong Kong, China

## Abstract

The clinical usefulness of pretreatment imaging techniques for predicting neck control in patients with oropharyngeal or hypopharyngeal squamous cell carcinoma (OHSCC) treated with chemoradiation remains unclear. In this prospective study, we investigated the role of pretreatment dynamic contrast-enhanced perfusion MR imaging (DCE-PWI), diffusion-weighted MR imaging (DWI), and [^18^F]fluorodeoxyglucose-positron emission tomography (^18^F-FDG PET)/CT derived imaging markers for the prediction of neck control in OHSCC patients treated with chemoradiation. Patients with untreated OHSCC scheduled for chemoradiation between August, 2010 and July, 2012 were eligible for the study. Clinical variables and the following imaging parameters of metastatic neck lymph nodes were examined in relation to neck control: transfer constant, volume of blood plasma, and volume of extracellular extravascular space (*V_e_*) on DCE-PWI; apparent diffusion coefficient (ADC) on DWI; maximum standardized uptake value, metabolic tumor volume, and total lesion glycolysis on ^18^F-FDG PET/CT. There were 69 patients (37 with oropharynx SCC and 32 with hypopharynx SCC) with successful pretreatment DCE-PWI and DWI available for analysis. After a median follow-up of 31 months, 25 (36.2%) participants had neck failure. Multivariate analysis identified hemoglobin level <14.3 g/dL (*P* = 0.019), *V_e_* <0.23 (*P* = 0.040), and ADC >1.14×10^−3^ mm^2^/s (*P* = 0.003) as independent prognostic factors for 3-year neck control. A prognostic scoring system was formulated by summing up the three significant predictors of neck control. Patients with scores of 2–3 had significantly poorer neck control and overall survival rates than patients with scores of 0–1. We conclude that hemoglobin levels, *V_e_*, and ADC are independent pretreatment prognostic factors for neck control in OHSCC treated with chemoradiation. Their combination may identify a subgroup of patients at high risk of developing neck failure.

## Introduction

Oropharyngeal and hypopharyngeal squamous cell carcinomas (OHSCC) are head and neck malignant tumors that originate from adjacent areas. These neoplasms share both similar lymphatic drainage and treatment regimens. Most patients with OHSCC have malignant cervical adenopathy at presentation and are treated with an organ-preservation approach based on chemoradiation [1]. Unfortunately, such treatment modality is not always successful, and approximately 20% of patients with OHSCC will develop neck failure [2], [3]. Early prediction of neck failure may allow for therapeutic modification, including the selection of suitable candidates for surgery and intensification of chemoradiation [3], [4]. In this scenario, the identification of reliable imaging biomarkers of neck control holds great promise for an improved risk stratification of patients undergoing chemoradiation.

Currently, diffusion-weighted MR imaging (DWI) and dynamic contrast-enhanced perfusion weighted imaging (DCE-PWI) are clinically feasible imaging methods for assessing the functional aspects of head and neck squamous cell carcinomas (HNSCC). DWI is a rapid MRI technique that allows quantification of the diffusion of water molecules in tissues using the apparent diffusion coefficient (ADC). Previous studies demonstrated that tumors with high ADC values are less likely to respond to chemoradiation [5], [6], possibly because a high ADC value may reflect the presence of micronecrosis and, consequently, increased resistance to the delivery of cytotoxic drugs as well as oxygen during chemoradiation. DWI has been used in the prediction of response to chemoradiation in HNSCC, but conflicting results have been reported [6]–[11].

DCE-PWI is a functional MRI technique which is based on sequential imaging acquired during the passage of a contrast agent through the tissue of interest. It can probe the tumor microvascular environment, including perfusion, the permeability of blood vessels and the volume of the extracellular space [12], [13], which can in turn be related to tumor radiosensitivity [14]. DCE-PWI has shown promise for predicting response to chemoradiation in HNSCC [3], [6], [8], [15], [16]. However, most of the previous study series were composed of patients with tumors originating from various head and neck sites, which may be characterized by different tumor biology. A study specifically focusing on OHSCC patients demonstrated that DCE-PWI of the primary tumor may help predict local control in OHSCC patients treated with chemoradiation [8]. However, treatment outcomes in patients with HNSCC are dependent both on the biology of the primary tumors and the metastatic nodes. Consequently, the different characteristics between the primary tumor and the affected nodes need to be assessed separately [17]. However, the prognostic value of nodal DCE-PWI in the prediction of neck control in patients with OHSCC treated with chemoradiation has not been investigated yet.

Clinical variables (e.g., hemoglobin levels, N-stage, and nodal volume) as well as [^18^F]Fluorodeoxyglucose-positron emission tomography (^18^F-FDG PET)/CT parameters, including the maximum standardized uptake value (SUV_max_), metabolic tumor volume (MTV), and total lesion glycolysis (TLG) have been used for predicting response to chemoradiation in head and neck tumors, albeit with discrepant results [2], [7], [18]–[28]. In this scenario, we designed the current prospective study to determine whether clinical variables and pretreatment DCE-PWI, DWI, ^18^F-FDG PET/CT-derived parameters can predict neck control in OHSCC patients treated with chemoradiation.

## Patients and Methods

### Ethics Statement

The Institutional Review Board of the Chang Gung Memorial Hospital approved the study protocol (protocol no. 98-3582B) in December 2009. All procedures followed the tenets of the Declaration of Helsinki, and written informed consent was obtained from all participants.

### Patients

Patients with newly diagnosed OHSCC scheduled for chemoradiation with curative intent were eligible for this prospective study. The study participants underwent a thorough pretreatment imaging evaluation that included conventional MRI, DWI, DCE-PWI, and ^18^F-FDG PET/CT. Inclusion criteria were as follows: (1) presence of biopsy-proven OHSCC, (2) presence of neck nodes of their shortest axial diameters greater than 10mm on MR images either with strong uptake on ^18^F-FDG PET/CT or with positive cytology findings on ultrasound-guided fine needle aspiration, (3) ability to provide written informed consent, and (4) no contraindications to MRI. Patients with a history of previous head or neck cancers, second malignancies, distant metastases, or renal failure were excluded.

### Treatment and Follow-up

The study participants received intensity-modulated radiotherapy with a 6-MV X-ray at 2 Grays (Gy) per fraction, with five fractions per week. The radiotherapy dose was 46−50 Gy for all subclinical risk areas, including the neck lymphatics, and 72 Gy for the primary tumor and affected lymph nodes. Concurrent chemotherapy consisted of cisplatin 50mg/m^2^ on day 1, oral tegafur 800mg/day plus leucovorin 60mg/day from day 1 to day 14. This scheme was repeated every two weeks through the radiotherapy course [29]. Following treatment, all patients underwent a routine clinical follow-up examination every 1 to 3 months. A follow-up MRI was performed at 3 months after completion of the treatment, and an additional MRI or CT scan was performed every 6 months thereafter or in presence of clinical deterioration. Surgery or ultrasound-guided fine needle aspiration was performed for any suspicious neck recurrences. Patients without pathologically-proven recurrence were followed up for at least 12 months after treatment or until death.

### MRI with DWI and DCE-PWI

MR imaging study was performed using a 3 Tesla MR scanner (Magnetom Trio with TIM, Siemens, Erlangen, Germany). Conventional MRI of the head and neck region were performed in the axial and coronal projections using the following sequences: T2-weighted turbo spin echo (TSE) with fat saturation; T1-weighted TSE; and postcontrast fat-saturated T1-weighted TSE. For the transverse images, 5-mm section thickness was acquired with a coverage extending from the nasopharynx to the supraclavicular region. DWI was acquired using single shot spin-echo echo-planar imaging with a modified Stejskal-Tanner diffusion gradient pulsing scheme. Motion-probing gradients with a b-value of 800s/mm^2^ were applied along three orthogonal directions. The imaging slice and coverage were identical for T1- and T2-weighted images. The repetition time (TR) and echo time (TE) were 8,200 ms and 84ms, respectively. The acquisition time was 2min and 28sec.

DCE- PWI was acquired using a 3D T1-weighted spoiled gradient-echo sequence with the following parameters: TR/TE = 3.5/1.13 ms, 230×230-mm field of view, and 108×128 matrix. The imaging slice and coverage were identical with the conventional T1- and T2-weighted images. A spatial saturation slab was implanted below the acquired region to minimize the inflow effect from the carotid arteries. Before the administration of the contrast agent, the baseline longitudinal relaxation time (T1_0_) values were calculated from images acquired with different flip angles (4°, 8°, 15°, and 25°). The dynamic series involved the use of the same sequence with a 15 flip angle. After four acquisitions of the dynamic baseline scanning, a standard dose (0.1 mmol/kg body weight) of gadopentetate dimeglumine (Gd-DTPA; Magnevist, Bayer-Schering, Burgess Hill, UK) was administered by a power injector through a cannula placed in the antecubital vein at a rate of 3 mL/s and immediately followed by a saline flush. A total of 80 volumes were acquired with a temporal resolution of 3.3 s. The acquisition time was 4min and 24sec.

### PET/CT Imaging

PET/CT scans were performed using a Discovery ST 16 integrated PET/CT system (GE Healthcare, Milwaukee, WIUSA). Patients were required to fast for at least 6h before examination. Before PET acquisition, helical CT was performed from the head to the proximal thigh. The following parameters were used: collimation 16×3 mm, tube rotation time 0.5 s, pitch 1.5, table speed 35 mm/s, 100 mA, 100 kVp. The PET emission images were obtained between 50 and 70min after injection of 18F-FDG (370 MBq) in the two-dimensional mode, with 3-min scanning time per table position. The PET emission data were reconstructed using CT scans for attenuation correction. Reconstruction was performed with the ordered subsets expectation maximization (OSEM) method, with 10 subsets and 4 iterations.

### Data Analysis

Analyses of DWI and DCE-PWI were performed on neck nodes of their shortest axial diameters greater than 10mm on MR images either with strong uptake on ^18^F-FDG PET/CT or with positive cytology findings on ultrasound-guided fine needle aspiration. ADC maps were reconstructed on a pixel-by-pixel basis. The ADC was measured on ADC maps by drawing the region of interest (ROI) on the largest neck node by an experienced head and neck radiologist (having more than 20 years of experience), with the aid of the T2-weighted MR images and the T1-weighted post-contrast MR images to avoid cystic or necrotic areas.

DCE-PWI analysis was performed using MATLAB 7.0 (The Mathworks, Natick, MA, USA). The signal intensities of the DCE-PWI data were converted from the contrast agent concentration by solving the nonlinear relationship between the signal intensity and contrast agent concentrations [30]. The extended Kety model [13] was used for the pharmacokinetic analysis in a voxel-wise manner. The arterial input function was extracted using the blind source separation algorithm [31]. ROIs were manually drawn in the largest neck node on DCE MR images by the radiologist in a manner similar to that described for the DWI analysis. The following pharmacokinetic parameters were collected: the volume transfer rate constant (*K^trans^*), relative extravascular extracellular space (*V_e_*) and relative vascular plasma volume (*V_p_*) as well as the efflux rate constant (*K_ep_*) which equals the ratio of *K^trans^* to *V_e_*.

The N-stage was recorded according to the 2010 cancer staging system as revised by the American Joint Committee on Cancer. The nodal gross tumor volume (GTV) of the affected neck lymph nodes was calculated with a CT-based three-dimensional radiation treatment planning system. The SUV and the MTV of neck nodal metastases were measured from attenuation-corrected ^18^F-FDG PET images using the PMOD software (PMOD Technologies Ltd, Zurich, Switzerland). The boundaries were drawn largely enough to include all of the affected neck nodes. An SUV threshold of 2.5 was used for the delineation of MTV [21], [32]. The contour of the target lesion inside the boundaries was automatically produced and the voxels presenting SUV intensity >2.5 within the contouring margin were incorporated to define MTV. The SUV, MTV, and TLG values of the target lesions were automatically determined by the software. The TLG was calculated as the product of the lesion mean SUV and the MTV.

### Outcome determination and statistical analysis

Neck control was measured from the first day of treatment to the time of neck failure or the date of the last follow-up. Neck failure was determined by histological confirmation (aspiration, biopsy, or surgical resection) in all participants. We used logistic regression analyses to identify the relationship between the 3-year neck control rates and the following baseline variables: pretreatment hemoglobin levels; N-stage; tumor location; GTV of the affected nodes; nodal SUV_max_, MTV, and TLG on ^18^F-FDG PET/CT; nodal ADC on DWI; and nodal *K^trans^*, *V_e_*, *V_p_* and *K*_ep_ on DCE-PWI. The neck control rates were plotted using the Kaplan-Meier method. The optimal cutoff values for both pretreatment clinical and imaging variables were determined using the log-rank test using the 3-year neck control rate observed in the entire study cohort [19], [33]. All of the prognostic variables identified by univariate analysis were entered into the multivariate model. Multivariate analysis was performed using the Cox proportional hazards model with a forward selection procedure. The Spearman’s rank correlation coefficient was used to investigate the correlations among the variables. After complement of chemoradiation, the impact of local control over the neck control was also evaluated. All statistical analyses were performed using the SPSS software package (version 13.0; SPSS Inc., Chicago, IL, USA). Two-tailed *P* values <0.05 were considered statistically significant.

## Results

Between August, 2010 and July, 2012, a total of 92 OHSCC patients underwent pretreatment DWI, DCE-PWI, and^ 18^F-FDG PET/CT. Twenty-three patients were excluded from the analysis, 11 of whom had small or unevaluable (too necrotic) lesions, 8 had considerable artifact on DWI or PWI, and 4 were dead before the definite diagnosis of neck failure could be determined. Consequently, 69 patients were available for the analysis (5 females and 64 males; mean age, 52±9.17 years). Thirty-nine of the 69 patients have been included in a previous investigation [8] with different research goals. Table 1 shows the general characteristics of the study participants. After a median follow-up time of 31 months (range, 7–49 months), 44 (63.8%) of the 69 patients achieved neck control, whereas the remaining 25 (36.2%) patients had neck failure. Of this patient group, 40 (58.6%) were alive and 29 (41.4%) were dead at the time of analysis. The 3-year neck control rate and overall survival rates were 63% and 65%, respectively.

**Table 1:** Baseline characteristics of our 69 OHSCC patients.

Characteristics	Entire cohort (n = 69)
**Age (year), median (range)**	50 (39–78)
**Sex, n (%)**	
Male	64 (92.8)
Female	5 (7.2)
**Hb value (g/dL), median (range)**	14.00 (6.10–17.20)
**Subsite, n(%)**	
Oropharynx	37 (53.6)
Hypopharynx	32 (46.4)
**N status, n(%)**	
N1	6 (8.7)
N2b	35 (50.7)
N2c	16 (23.2)
N3	12 (17.4)
**Stage, n(%)**	
III	3 (4.3)
IVA	50 (72.5)
IVB	16 (23.2)
**GTV (cm^3^), median (range)**	9.90 (0.70–173.90)
***K^trans^*** ** (min** ^−**1**^ **), median (range)**	0.49 (0.03–1.62)
**V** ***_p_*** ** (× 10^3^), median (range)**	2.40 (0.01–185.10)
**V** ***_e_*** **, median (range)**	0.18 (0.01–0.69)
**K** ***_ep_*** ** (min** ^−**1**^ **), median (range)**	3.08 (0.16–20.70)
**ADC (× 10** ^−**3**^ **mm^2^/s), median (range)**	0.95 (0.46–1.28)
**SUV, median (range)**	11.13 (2.90–23.05)
**MTV (cm^3^), median (range)**	12.77 (0.16–135.30)
**TLG, median (range)**	62.88 (0.43–966.95)

**Figure 1:**
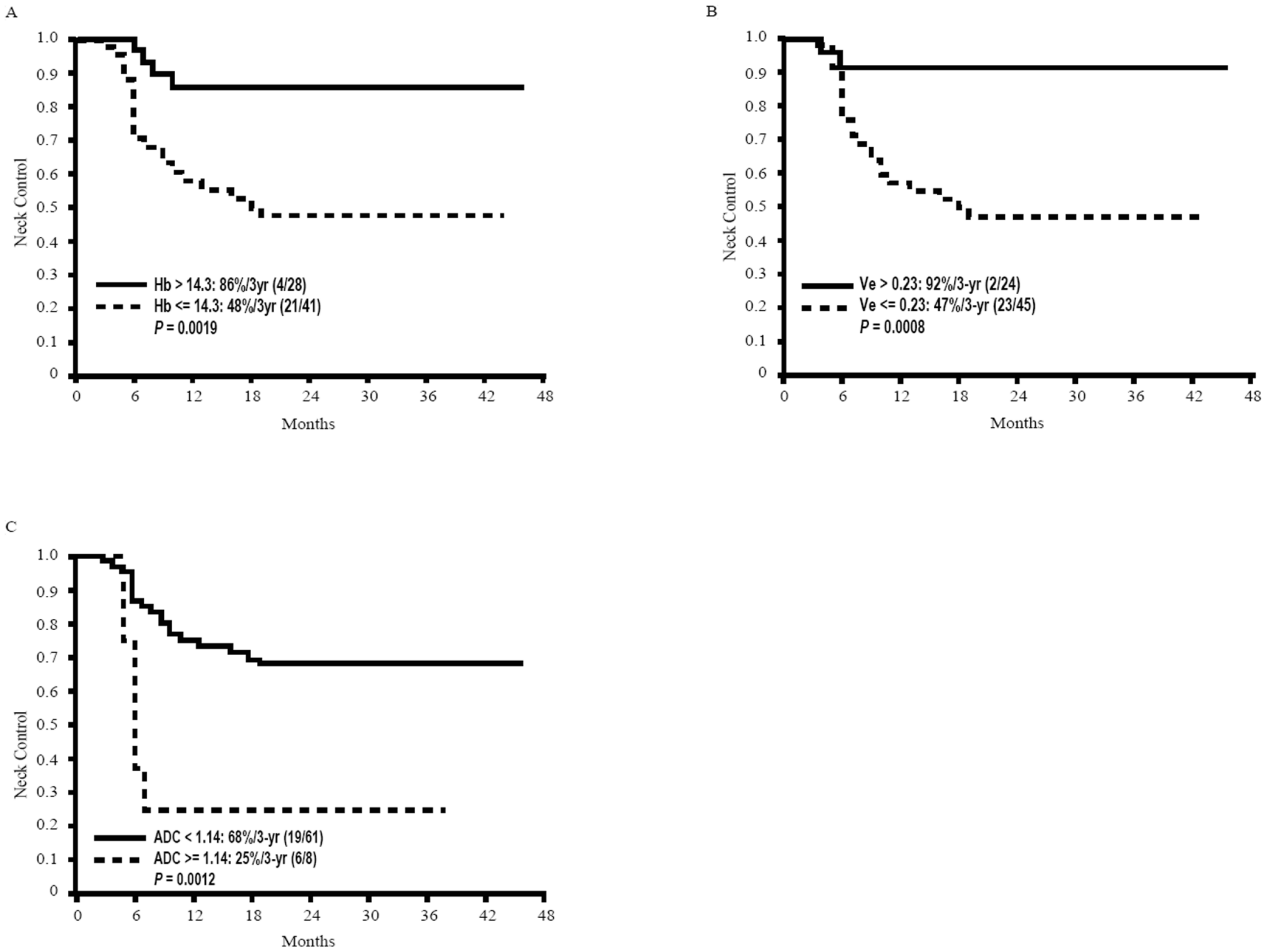
Kaplan-Meier estimates of neck control rates stratified according to hemoglobin level, ADC, and *V*_e_ values.

**Figure 2:**
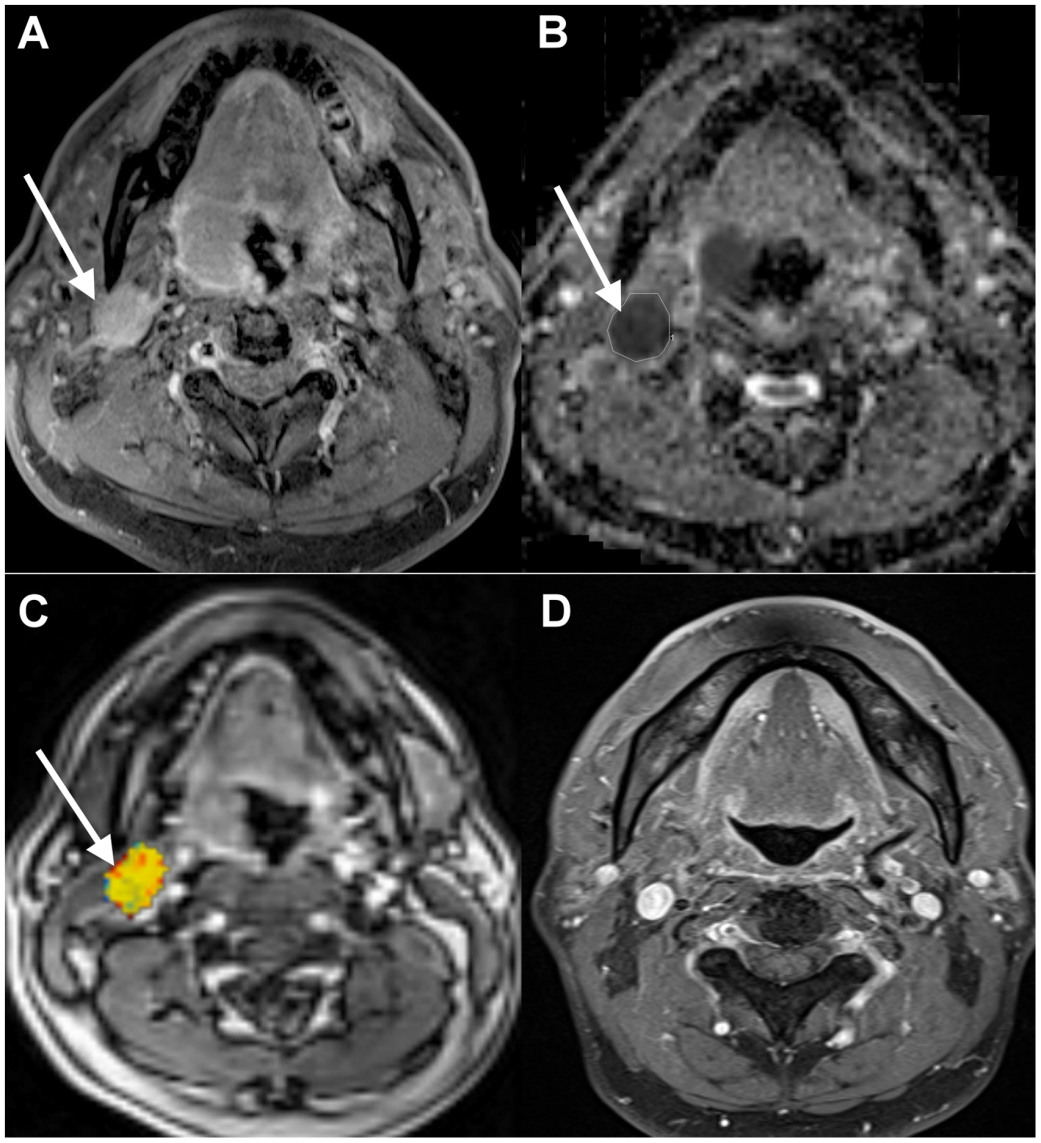
Neck control in a 53-year-old male patient with right tonsillar oropharyngeal SCC after chemoradiation. The patient’s hemoglobin level was 15.9 g/dL. A. Pretreatment axial-enhanced MRI identified a right cervical metastatic node (arrow). B. The corresponding ADC map showed a nodal ADC value of 0.78×10^−3^ mm^2^/s. C. The corresponding PWI map showed a nodal *V*_e_ value of 0.34. D. Post-treatment axial-enhanced MRI demonstrated a complete regression of the right metastatic node. A 15-month clinical and imaging follow-up did not disclose any nodal recurrence.

**Figure 3:**
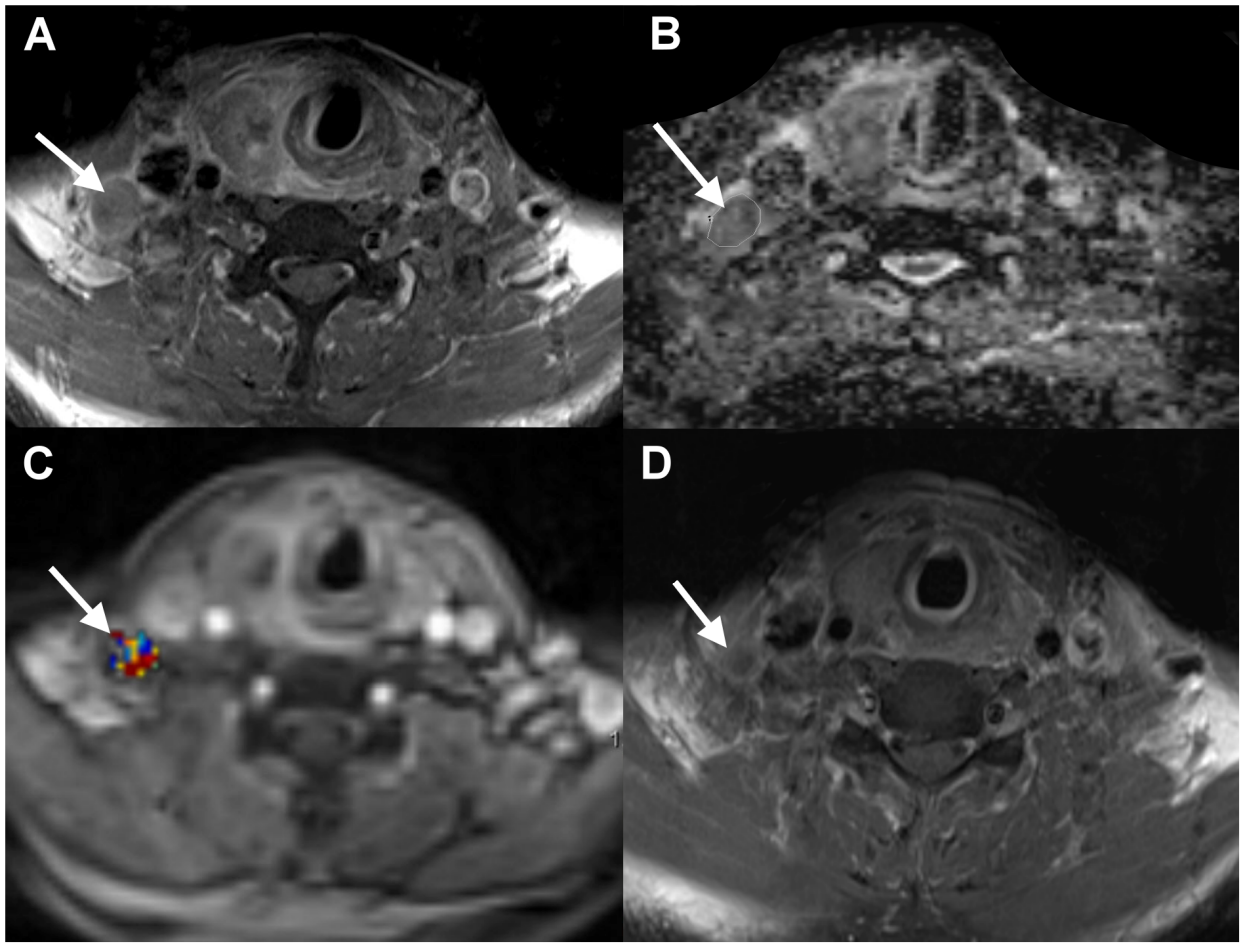
Neck failure in a 45-year-old male patient with right hypopharyngeal SCC after chemoradiation. The patient’s hemoglobin level was 12.7 g/dL. A. Pretreatment axial-enhanced MRI identified a right cervical metastatic node (arrow). B. The corresponding ADC map showed a nodal ADC value of 1.24×10^−3^ mm^2^/s. C. The corresponding PWI map showed a nodal *V*_e_ value of 0.07. D. Post-treatment axial-enhanced MRI demonstrated a residual right cervical node (arrow), which was further confirmed by neck dissection.

Univariate and multivariate analyses were carried out to identify significant prognostic factors in the entire study cohort (Table 2). The results of univariate analysis identified a hemoglobin level <14.3 g/dL (*P* = 0.0019), nodal GTV >14.6 cm^3^ (*P* = 0.0019), *K^trans^* <0.84 min^−1^(*P* = 0.0163), Ve <0.23 (*P* = 0.0008), ADC >1.14×10^−3^ mm^2^/s (*P* = 0.0012), SUV >6.25 (*P* = 0.0438), MTV >38.05 cm^3^ (*P* = 0.0148), and TLG >217.18 (*P* = 0.0019) as significant predictors of the 3-year neck control rate. After allowance for potential confounders, hemoglobin level (*P* = 0.0019), *V_e_* (*P* = 0.040), and ADC (*P* = 0.003) were found to be independent predictors of neck control in multivariate analysis (Figs. 1−3).

**Table 2:** Univariate and multivariate analyses of 3-year neck control rates in OHSCC patients (n = 69).

	Univariate analysis		Multivariate analysis**	
Characteristics (n, %)	Neck control (3-year %, n event)	*P*	HR (95% CI)	*P*
**Age (years)**		0.7616		ns
<65 (62, 89.9)	63 (23)			
≥65 (7, 10.1)	69 (2)			
**Sex**		0.4896		ns
Male (64, 92.8)	62 (24)			
Female (5, 7.2)	80 (1)			
**Hemoglobin (g/dL)** *		0.0019	3.66 (1.24–10.84)	0.019
>14.30 (28, 40.6)	86 (4)			
≤14.30 (41, 59.4)	48 (21)			
**Subsites**		0.8354		ns
Oropharynx (37, 63.6)	62 (14)			
Hypopharynx (32, 46.4)	64 (11)			
**GTV (cm^3^)** *		0.0301		ns
<14.60 (42, 60.9)	73 (11)			
≥14.60 (27, 39.1)	48 (14)			
**N status** *		0.1152		ns
N1-2b (41, 59.4)	70 (12)			
N2c-3 (28 40.6)	53 (13)			
**Stage** *		0.1709		ns
III-IVA (53, 76.8)	67 (17)			
IVB (16, 23.2)	50 (8)			
**K** ***^trans^*** ** (min** ^−**1**^ **)** *		0.0163		ns
>0.84 (14, 20.3)	93 (1)			
≤0.84 (55, 79.7)	55 (24)			
**V** ***_p_*** ** (× 10^3^)** *		0.0809		ns
>1.30 (38, 55.1)	73 (10)			
≤1.30 (31, 44.9)	51 (15)			
**V** ***_e_*** *		0.0008	4.67 (1.07–20.33)	0.040
>0.23 (24, 34.8)	92 (2)			
≤0.23 (45, 65.2)	47 (23)			
**K** ***_ep_*** ** (min** ^−**1**^ **)** *		0.1539		ns
<4.91 (48, 69.6)	68 (15)			
≤4.91 (21, 30.4)	52 (10)			
**ADC (× 10** ^−**3**^ **mm^2^/s)** *		0.0012	5.14 (1.77–14.93)	0.003
<1.14 (61, 88.4)	68 (19)			
≥1.14 (8, 11.6)	25 (6)			
**SUV** *		0.0438		ns
<6.25 (8, 11.6)	100 (0)			
≥6.25 (61, 88.4)	58 (25)			
**MTV (cm^3^)** *		0.0148		ns
<38.05 (52, 75.4)	70 (15)			
≥38.05 (17, 24.6)	41 (10)			
**TLG** *		0.0065		ns
<217.18 (55, 79.7)	70 (16)			
≥217.18 (14, 20.3)	36 (9)			

*Optimal cutoff value for each parameter.

**All of the factors identified in univariate analysis were entered into the multivariate model. Only significant values were listed in the Table.

HR indicates hazard ratio; CI, confidence interval; ns, not significant.

**Figure 4:**
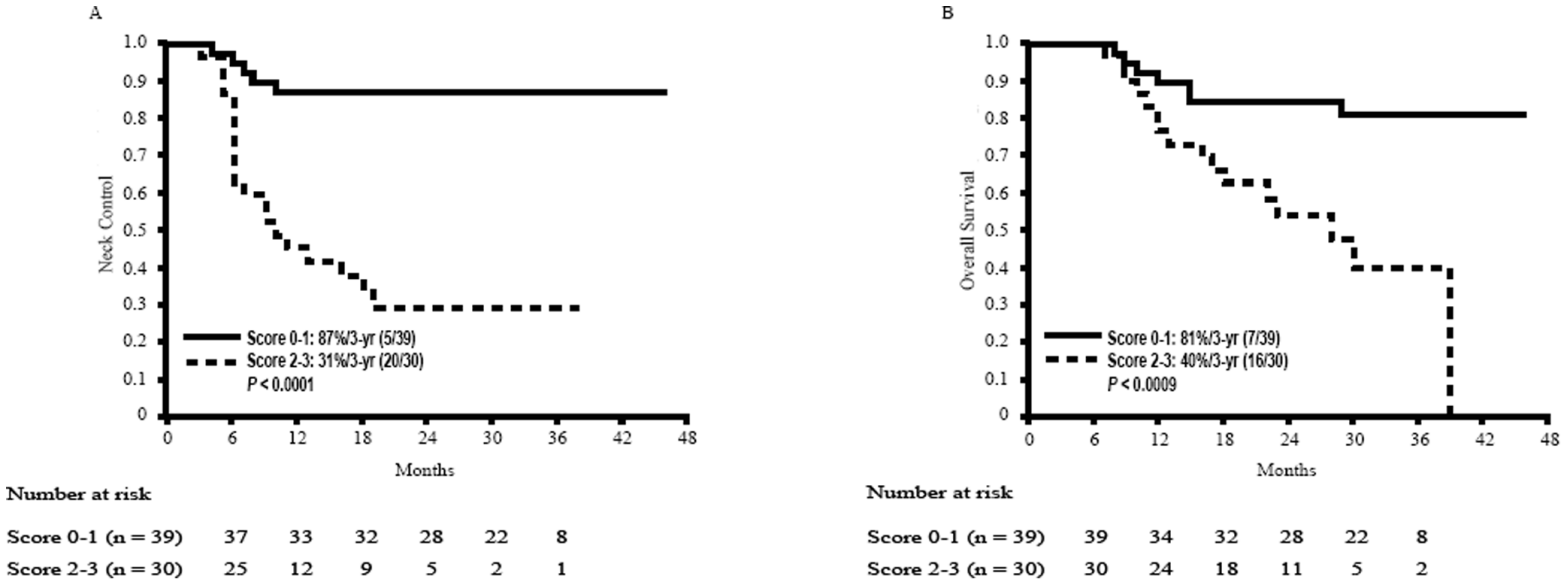
Kaplan-Meier estimates of 3-year neck control rates and overall survival in OHSCC patients according to the prognostic scoring system with combination of pretreatment hemoglobin level, ADC, and *V*_e_ values.

Spearman correlation analysis showed significant correlations between *K^trans^* and *V_e_* (*P*<0.01) and between *V_e_* and *Vp* (*P*<0.05). Correlations at a *P*<0.01 level were also noted for nodal GTV and SUV_max_, MTV and TLG, SUV_max_ and MTV, SUV_max_ and TLG, as well as MTV and TLG. Hemoglobin level and ADC values did not correlate with any of the DCE-PWI or ^18^F-FDG PET/CT parameters. Since no significant correlations were found between hemoglobin, *V_e_*, and ADC, we further assessed whether these independent factors can have a complementary role for the prognostic stratification of OHSCC patients. A prognostic scoring system was formulated by summing up the three significant pretreatment covariates: 0 for hemoglobin level >14.3 g/dL and 1 for hemoglobin level <14.3 g/dL; 0 for *V_e_*>0.23 and 1 for *V_e_*<0.23; 0 for ADC <1.14×10^−3^ mm^2^/s and 1 for ADC >1.14×10^−3^ mm^2^/s. The 3-year neck control rates were 100% for patients with a score of 0 (absence of all of the three independent risk factors), 82% for patients with a score of 1 (presence of one independent risk factor), 34% for patients with a score of 2 (presence of two independent risk factors), and 17% for patients with a score of 3 (presence of all of the three independent risk factors). The 3-year neck control rate of patients with scores of 0–1 was 56% higher than that of patients with scores of 2–3 (87% vs. 31%, *P*<0.0001). Moreover, the 3-year overall survival rate of patients with scores of 0–1 was 41% higher than that of patients with scores of 2–3 (81% vs. 40%, *P*<0.0009) (Fig. 4). Multivariate Cox proportional hazard analysis demonstrated that patients with scores of 2–3 had significantly poorer neck control (*P*<0.001, HR [95% CI]: 7.49 [2.79–20.12]) and overall survival (*P* = 0.002, HR [95% CI]: 4.17 [1.68–10.34]) rates than patients with scores of 0–1 (Table 3).

**Table 3:** Multivariate analyses of 3-year neck control and overall survival rates according to the prognostic scoring system based on hemoglobin levels, *V_e_*, and ADC.

Score	Neck control	Overall survival
	*P*, HR (95% CI)	*P*, HR (95% CI)
0–1	Reference	Reference
2–3	<0.001, 7.49 (2.79–20.12)	0.002, 4.17 (1.68–10.34)

HR indicates hazard ratio; CI, confidence interval.

**Figure 5:**
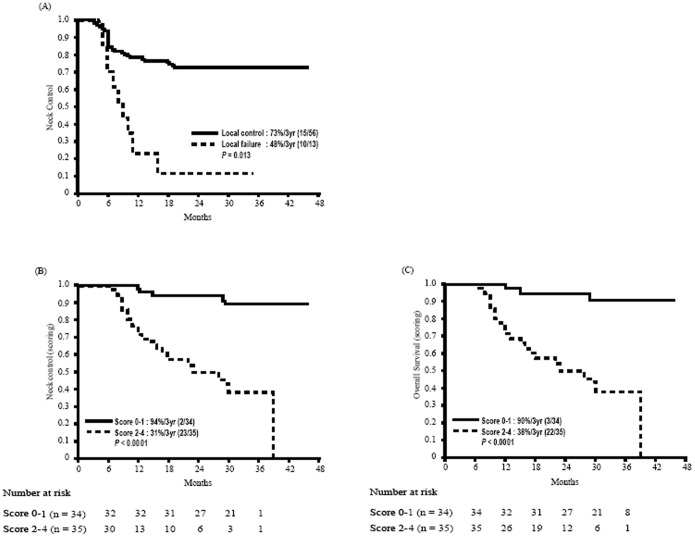
A. Kaplan-Meier estimates of neck control rates stratified according to the presence of local failure. B and C, Kaplan-Meier estimates of 3-year neck control rates and overall survival in OHSCC patients according to the prognostic scoring system based on pretreatment hemoglobin level, ADC, V_e_ values, and posttreatment local control status.

Of the 69 study patients, 13 had local failure (of whom ten had both local and neck failures, whereas the remaining three had local failure alone). Univariate analysis demonstrated that local control was significantly associated with the 3-year neck control rate (P = 0.013; Fig. 5A). Notably, this association persisted even after allowance for potential confounders in multivariate analysis (P = 0.019). After the addition of this post-treatment factor to our prognostic scoring system, we found that the 3-year neck control rate of patients with scores of 0–1 was 63% higher than that of patients with scores of 2–4 (94% vs. 31%, P<0.0001). Moreover, the 3-year overall survival rate of patients with scores of 0–1 was 52% higher than that of patients with scores of 2–4 (90% vs. 38%, P<0.0001; Fig. 5B, C).

## Discussion

This prospective study evaluated whether imaging parameters and other clinical variables could predict neck control in OHSCC patients undergoing chemoradiation. Among the DCE-PWI-derived parameters, we found that pretreatment nodal *K^trans^* and *V_e_* were significantly associated with neck control in univariate analysis. However, only nodal *V_e_* remained a significant independent prognostic factor after allowance for potential confounders in multivariate analysis. *K^trans^* is a pharmacokinetic parameter that reflects lesion vascularity and permeability, which can in turn reflect the delivery of oxygen and chemotherapy drugs during chemoradiation [34]. In our previous study conducted in 58 OHSCC patients treated with chemoradiation [8], we found that *K^trans^* of the primary tumor was the only DCE-PWI-derived parameter associated with local control. However, a study performed in 24 patients with HNSCC (comprising tumors of the tongue, larynx, and oropharynx) failed to demonstrate such an association [6]. Therefore, the value of primary tumor *K^trans^* for predicting local response may vary among different tumor types. In contrast, nodal *K^trans^* has been reported to be the only DCE-PWI parameter that could predict nodal response to chemoradiation in various forms of head and neck cancers [3], [6], [15]. Although *K^trans^* in this study was significantly associated with neck control in univariate analysis, it did not retain its independent prognostic significance in multivariate analysis as *V_e_* did. Consequently, *V_e_* appeared to be a better DCE-PWI predictor of neck control than *K^trans^* in OHSCC patients treated with chemoradiation.

*V_e_* is a DCE-PWI-derived pharmacokinetic parameter that reflects the extravascular extracellular space. Compared with normal tissues, the tumor extracellular space is characterized by a larger interstitial space, higher collagen concentration, higher interstitial fluid pressure, and higher effective interstitial diffusion coefficient of macromolecules [35]. In a prospective study of patients with osteosarcoma treated with chemotherapy, Guo *et al.* [36] reported that *V_e_* and *K^trans^* were significant predictors of treatment outcomes. However, previous studies conducted in patients with head and neck malignancies [3], [6], [15] failed to identify significant differences in *V_e_* values between responders and nonresponders. The association between neck control and pretreatment nodal *V_e_* observed in our OHSCC patients is in line with the results obtained by Guo *et al.* [36]. Taken together, these findings suggest that the nodal extravascular extracellular space may play a role in the prediction of response to chemoradiation. Another important finding in this study is the significant positive correlation between *K^trans^* and *V_e_* values measured in neck node metastases originating from OHSCC. These results are in line with the previously reported positive correlations of *K^trans^* and *V_e_* in gliomas [37], [38].

DWI allows quantification of the diffusion of water molecules in tissues microstructure by using ADC, which is inversely correlated with cell density [39]. Although some DWI studies in patients with head and neck cancer have demonstrated that pretreatment ADC can be used as a potential marker for prediction of local failure [7], [10] and neck failure [11], other reports failed to identify such an association [6], [8], [9]. Several factors may at least in part explain such discrepancies, including different tumor types, sample sizes, and treatment protocols. In our previous study of OHSCC patients, pretreatment ADC values of the primary tumor did not predict local response to chemoradiation. In contrast, the current study demonstrates that pretreatment ADC values of the neck metastatic nodes were an independent prognostic factor for neck control. Our data strengthen the notion that actively proliferating solid tumors (characterized by a lower ADC) are more likely to have a better perfusion than those with a low cellularity (characterized by a higher ADC), facilitating a better delivery of oxygen and cytotoxic drugs during chemoradiation [11].

^18^F-FDG PET/CT is commonly used for the staging workup of OHSCC patients because of its clinical value in identifying subcentimeter nodal metastases, distant metastases, and second primary cancers [40]. FDG PET can provide three parameters, i.e., SUV (that reflects glucose metabolism), MTV (that reflects tumor burden), and TLG (that integrates both glucose metabolism and tumor burden). The prognostic significance of pretreatment nodal FDG PET parameters in patients with head and neck malignancies remains controversial. Demirci et al [23] demonstrated that nodal SUV was a significant predictor of disease-free survival. In patients with oral cavity cancer, Liao et al [25] found that nodal SUV predicted worse outcomes. Kubicek et al [26] reported that nodal SUV was significantly associated with distant recurrences but not with locoregional control. However, other studies failed to identify an association between nodal SUV, nodal MTV, or nodal TLG with treatment outcomes [22], [24], [27], [28]. A major source of confounding that may explain at least in part such discrepancies is the inclusion of tumors originating from various head and neck sites. In this study with primary OHSCC uniformly treated with chemoradiation, although all of the nodal FDG PET/CT parameters were statistically significant prognostic factors for neck control in univariate analysis, they did not retain their independent prognostic significance in multivariate analysis. Based on our results, the use of nodal FDG PET parameters cannot be currently recommended for the prediction of neck control in OHSCC patients treated with chemoradiation. However, larger prospective studies focusing on specific tumor sites are needed to allow more definite conclusions.

The pretreatment hemoglobin level is a well-known predictor of chemoradiation efficacy in patients with head and neck cancer [41]–[43], possibly via modulation of tumor oxygenation. An increase in hemoglobin level can increase oxygen levels and reduce the hypoxic fraction in head and neck malignancies, ultimately facilitating the cytotoxic effects of chemoradiation [41]–[43]. However, Ohnishi *et al.* [7] reported that pretreatment hemoglobin level was only marginally associated with local failure in a series of 64 OHSCC patients treated with chemoradiation. In our previous series of 58 OHSCC patients treated with chemoradiation, hemoglobin level did not show any significant association with local control. However, the present study confirmed the significance of hemoglobin level in predicting neck control in OHSCC patients after chemoradiation. Taken together, these results suggest that hemoglobin level may have different influences on the response of the primary tumor vs. those of metastatic nodes to chemoradiation among OHSCC patients.

We have previously shown that primary tumor location (oropharynx versus hypopharynx) among OHSCC patients was weakly associated with local control (*P* = 0.07) [8]. In the current study, however, the primary tumor location was not associated with neck control, suggesting that the biological microenvironment of metastatic lymph nodes from oropharyngeal SCC does not differ significantly from that of hypopharyngeal SCC. Consequently, the primary tumor location might not be a major determinant of nodal response to chemoradiation. Although N stage and nodal volume have been reported to be significantly associated with treatment outcomes in HNSCC patients after radiotherapy or chemoradiation [2], [19], [21], other studies failed to report such associations [3], [7], [11], [17], [20]. Our current results argue against the predictive role of N stage and nodal GTV for neck control in OHSCC patients treated with chemoradiation.

Most OHSCC patients have malignant cervical adenopathy at presentation and are currently treated with an organ-preservation approach based on chemoradiation. In this context, the identification of patients at high risk of treatment failure before institution of chemoradiation is paramount. In this study, 36% of OHSCC patients showed neck failure after chemoradiation. Our prognostic scoring system obtained by combining the three pretreatment predictors significantly improved the prediction of treatment outcomes. Specifically, patients bearing at least two risk factors were at high risk of developing neck failure and having poor survival. Consequently, these patients should be closely followed up after chemoradiation to ensure early detection of potentially salvageable lesions. Alternatively, they may serve as suitable candidates for planned neck dissection or novel treatment approaches. In this scenario, the use of multimodal imaging with both DCE-PWI and DWI may be justified in OHSCC patients. In addition, our results demonstrated that the posttreatment primary tumor status was significantly associated with nodal control. Moreover, local failure was an independent predictor of neck failure.

Some limitations of our study merit comment. First, since our OHSCC patients were treated by chemoradiation instead of surgery, we did not have histological confirmation for all selected lymph nodes. As this was not feasible either ethically or clinically, the absence of pathological proof for such target lesions is a common caveat inherent in this type of study. A shortest axial nodal diameter greater than 10 mm on MRI and/or a strong FDG uptake on ^18^F-FDG/PET-CT are well accepted clinical criteria for the presence of nodal metastasis. Notably, their combination (as used in this study) has a reported accuracy as high as 91.3% [44]. Second, DWI and DCE-PWI values are dependent on the choice of the ROI. In order to minimize potential biases, all of the ROIs in this study were drawn by an experienced radiologist. Finally, our series comprising OHSCC arising from the contiguous locations with similar lymphatic patterns and treatment regimens is relatively homogeneous compared with previous relevant imaging studies conducted on patients with head and neck cancers originating from various sites. Moreover, it can be argued that the response to chemoradiation of hypopharyngeal SCC may be different from that of oropharyngeal SCC, particularly in cases where human papillomavirus plays a causative role. Although complete data on human papillomarvirus infection were not available for this study, it is noteworthy that the nodal response observed in patients with oropharyngeal SCC was quite similar to those of patients with hypopharyngeal SCC.

## Conclusions

The results from the present study suggest that hemoglobin levels <14.3 g/dL, *V_e_* <0.23, and ADC >1.14×10^−3^ mm^2^/s are independent adverse pretreatment prognostic factors for neck control in OHSCC patients undergoing chemoradiation. Their combination may identify a subgroup of patients at high risk of developing neck failure. We conclude that these patients should be closely followed up after chemoradiation to ensure early detection of potentially salvageable lesions. Alternatively, they may serve as suitable candidates for surgery or novel treatment approaches.
